# Differential Regulation of Cholesterol-Related Gene Expression by HDAC Inhibition in Neuron-Enriched and Glia-Enriched Rat Primary Cultures

**DOI:** 10.3390/ijms27209231

**Published:** 2026-10-17

**Authors:** Alevtina A. Samorodova, Alexander P. Beletskiy, Pavel M. Balaban, Anastasia A. Borodinova

**Affiliations:** Laboratory of Cellular Neurobiology of Learning, Institute of Higher Nervous Activity and Neurophysiology, Russian Academy of Sciences, Moscow 117485, Russia

**Keywords:** Epigenetics, histone, HDAC inhibitor, metabolism, cholesterol, gene expression, neuron, glia, SREBP, LXR

## Abstract

Brain cholesterol homeostasis is a highly sophisticated, epigenetically regulated process where neurons and glia play distinct roles in maintaining metabolic balance. Disruption of this tightly regulated system is a hallmark of many neurological disorders, including Alzheimer’s disease. Histone deacetylase (HDAC) inhibitors were shown to regulate transcription of metabolic genes in the brain. However, their influence on cholesterol-related gene expression in functionally different neural cell populations remains insufficiently characterized. In this study, we used a model of rat primary cultures enriched in either neurons or glial cells to compare the culture-specific transcriptional responses induced by the HDAC inhibitor trichostatin A (TSA). Using quantitative PCR, we analyzed the relative mRNA expression of representative genes involved in cholesterol synthesis (*Hmgcs1*, *Mvd*, *Lss*, *Dhcr7*), transport (*Apoe*, *Apod*), uptake (*Vldlr*, *Ldlr*), efflux (*Abca7*, *Abcg1*), and elimination (*Cyp46a1*). Our results suggest that HDAC inhibition induces complementary yet distinct and epigenetically tunable transcriptional responses in the neuronal and glial cell cultures maintained under their respective culture-specific conditions. We hypothesize that these transcriptional changes may have functional consequences for cholesterol metabolic pathways, including altered cholesterol accumulation in both cell types and enhanced cholesterol efflux in neurons. However, this requires further experimental validation. Overall, our results provide a framework for understanding how cell-type-specific epigenetic regulation of cholesterol-related genes may shape brain lipid homeostasis. These insights may facilitate the development of cell-targeted epigenetic therapies for neurological disorders associated with metabolic dysfunction.

## 1. Introduction

Lipid homeostasis is essential for normal brain development and function, and its dysregulation increases with age, contributing to many neurological disorders [1,2,3,4,5,6]. Cholesterol is the most abundant sterol in the brain that serves both as a structural component of cellular membranes [7], and as a secretory molecule involved in neuroglial communication [5,8,9,10]. Because cholesterol from peripheral tissues cannot cross the blood–brain barrier [11], the brain maintains its own cholesterol pool through a coordinated activity of *de novo* synthesis from acetyl-CoA, receptor-mediated uptake, transporter-driven efflux, and metabolic elimination in neurons and glia, which are tightly regulated by sterol-sensing feedback mechanisms involving SREBP2 and LXR signaling [12,13,14,15].

Astrocytes are a type of glial cell that functions as the major metabolic hub in the brain and supplies neurons with cholesterol, which is essential for neuronal maturation, synaptogenesis, synaptic plasticity, learning and memory [4,5,8,9,16,17,18]. Neurons synthesize cholesterol inefficiently under physiological conditions, and rely predominantly on astrocyte-derived cholesterol [12,19], whereas during development or under pathological conditions neurons can increase cholesterol synthesis to support differentiation and repair [20,21]. Astrocyte-derived cholesterol is transported in apolipoprotein-containing lipoprotein particles, predominantly ApoE [8,22], which bind to specific cell-surface lipoprotein receptors and promote cholesterol uptake via receptor-mediated endocytosis [8,12]. In neurons, cholesterol supports membrane architecture and lipid rafts—highly organized membrane microdomains enriched in receptors, enzymes, and signaling molecules that are essential for efficient neurotransmission [7]. Cholesterol and its derivatives play important roles at both pre- and postsynaptic sites, regulating glutamate receptor signaling, exocytosis, synaptic transmission and neuronal excitability [9,17,18,23,24].

Excessive cholesterol can be directly exported from neurons and glial cells to extracellular lipid acceptors—apolipoproteins via specific membrane ATP-binding cassette (ABC) transporters [25,26,27]. Neurons also express cholesterol 24-hydroxylase CYP46A1, which converts free cholesterol to 24S-hydroxycholesterol to provide cholesterol clearance [28].

Neurons and glia maintain brain cholesterol homeostasis through bidirectional communications and coordinated regulation of gene expression. Culturing glia with neurons was shown to induce astrocytic expression of genes involved in cholesterol biosynthesis and lipid metabolism [10,29]. Astrocyte-derived ApoE particles, enriched with cholesterol and various microRNAs, can suppress neuronal expression of cholesterol synthesis enzymes, while concomitantly increasing expression of the synaptic plasticity-related genes [10,30,31]. Knockdown of the neuronal ApoE receptors LDLR or LRP1 disrupts neuronal cholesterol uptake and impairs this bidirectional neuroglial communication [10].

Numerous studies have linked dysregulated cholesterol metabolism to the development of neurological disorders, including Alzheimer’s disease, Parkinson’s disease, Huntington’s disease, and other forms of neuropathology characterized by synaptic dysfunction [1,3,6,18]. Alterations in cholesterol synthesis and distribution can change the abundance and composition of lipid rafts, thereby disrupting cellular signaling, synaptic transmission and increasing the neuronal excitability [18,23]. Evidence from non-neuronal and primary neuron cultures indicates that cholesterol-related genes can be regulated epigenetically by histone acetylation and microRNAs [14,30,32,33].

Among various approaches, histone deacetylase (HDAC) inhibition has emerged as a promising therapeutic technique for compensation of neurological disorders [34,35]. Multiple studies indicate that application of HDAC inhibitors can modulate cholesterol metabolism as a part of a complex cellular response in diverse cell types [14,32,36,37,38,39,40,41,42], and can reinstate the metabolic homeostasis in disease models [32,39,41].

However, although HDAC inhibition remains a promising therapeutic strategy for regulating lipid homeostasis under pathological conditions, the cholesterol-related gene networks targeted by HDAC inhibitors in specific brain cell types remain largely unexplored. To address this gap, we directly compared the transcriptional responses of rat neuron-enriched and glia-enriched cultures (see the Methods section for further details) to the HDAC inhibitor trichostatin A (TSA), focusing on genes involved in regulation of cholesterol homeostasis. qPCR analysis revealed a differential epigenetic regulation of cholesterol-related gene expression in primary neuron cultures and mixed glial cultures maintained under their respective culture-specific conditions. We hypothesized that these transcriptional changes may have functional consequences for cholesterol metabolic pathways, which require further experimental validation. These findings may facilitate the development of cell-targeted epigenetic therapies for neurological disorders associated with metabolic dysfunction.

## 2. Results

In previous studies, we demonstrated that pharmacological HDAC inhibition with sodium butyrate (NaB, 5 mM) or trichostatin A (TSA, 100 nM) induces widespread transcriptomic changes in primary cortical neuron cultures [43,44]. These changes were associated with altered brain cell differentiation, alternative splicing of synaptic-plasticity-related genes, and neural network activity—processes that are strikingly dependent on cellular metabolism.

**Figure 1:**
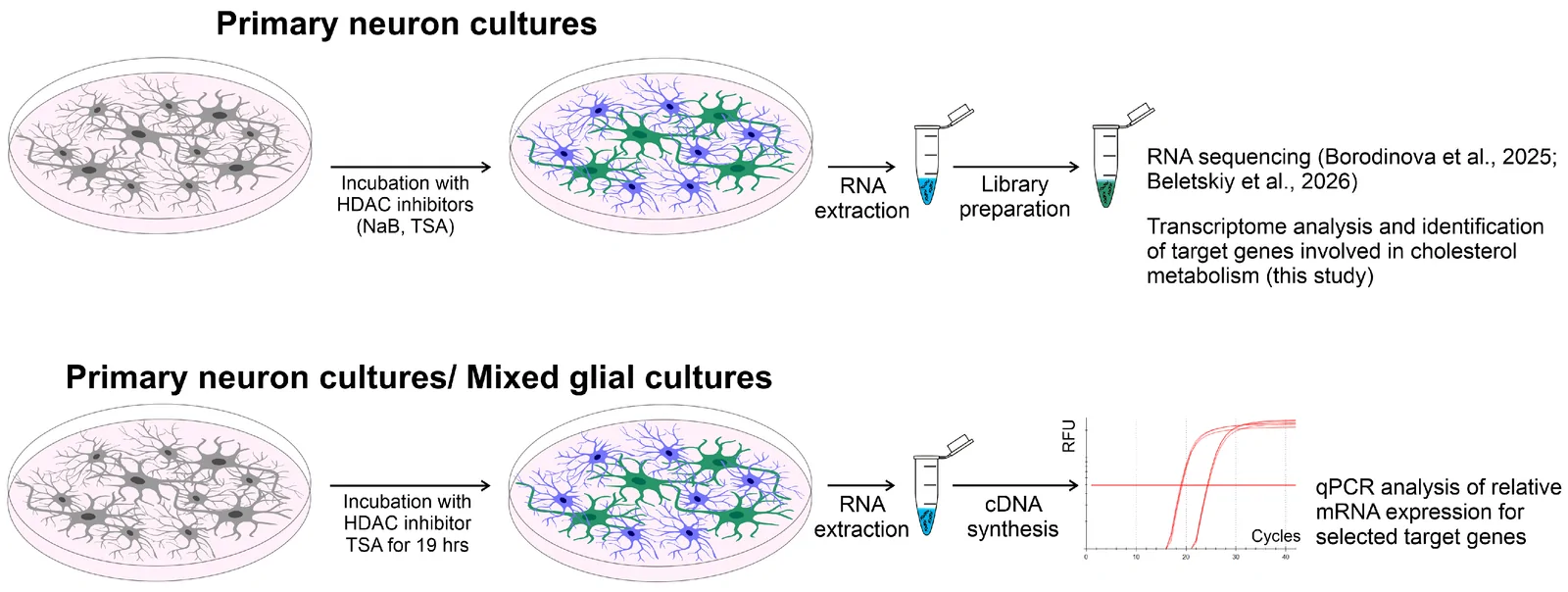
Experimental workflow for investigating the epigenetic regulation of cholesterol-associated gene networks in neuron-enriched primary neuron cultures and glia-enriched mixed glial cultures maintained under culture-specific conditions. The top panel shows the experiments that preceded the acquisition of RNA-seq data. RNA sequencing was performed previously [43,44], and the data were used in this study for Gene Ontology analysis and heatmap generation. The bottom panel shows the experiments conducted exclusively for this study. HDAC—histone deacetylase; NaB—sodium butyrate; TSA—trichostatin A; qPCR—quantitative (real-time) PCR.

These findings prompted us to investigate the potential epigenetic regulation of cholesterol-associated gene networks, which may contribute to the metabolic remodeling that is essential for cell differentiation and plasticity-related processes. We therefore re-analyzed the previously generated RNA-seq dataset [43] and identified the differentially expressed genes (DEGs) involved in the regulation of cholesterol homeostasis (Figure 1). Using this dataset, we selected the target genes for further investigation. Using neuron-enriched primary neuron cultures and glia-enriched mixed glial cultures maintained under culture-specific conditions (see the Methods section for further details), we examined cell type-specific responses to chromatin remodeling induced by HDAC inhibition, and assessed expression of the selected genes involved in cholesterol homeostasis by quantitative PCR (Figure 1). In addition, Western blot analysis of primary cortical neuron cultures confirmed that TSA increased histone acetylation, consistent with effective HDAC inhibition (Supplementary Figure S1).

### 2.1. RNAseq Analysis Revealed Epigenetic Regulation of Genes Involved in Cholesterol Homeostasis in Primary Neuron Cultures

**Figure 2:**
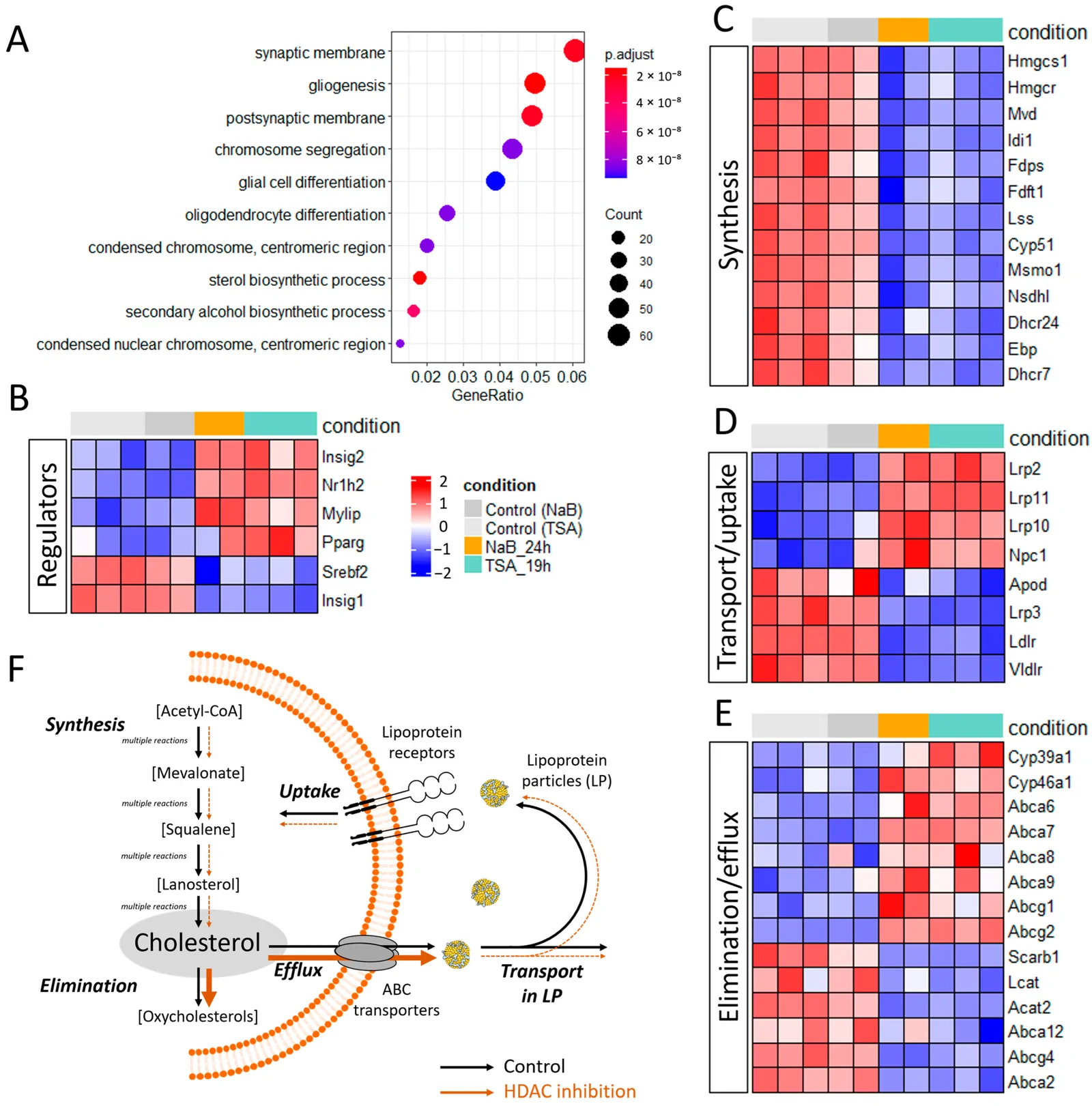
Prolonged HDAC inhibition using sodium butyrate (NaB) or trichostatin A (TSA) broadly remodels the cholesterol homeostasis transcriptome in rat primary cortical neuron cultures. (**A**)—Gene ontology analysis reveals significant enrichment of the “sterol biosynthetic process” term among downregulated differentially expressed genes (DEGs). (**B**–**E**)—Cluster analysis of DEGs involved in cholesterol synthesis, transport/uptake, elimination/efflux, and regulation of cholesterol homeostasis. Heatmap shows differential expression in NaB-treated (orange, *n* = 2) and TSA-treated neuron cultures (turquoise, *n* = 3), each compared to its own time-matched control cultures (gray; *n* = 2 for NaB, *n* = 3 for TSA). Values are shown as z-scored log-transformed normalized expression counts. The color scale indicates the expression levels (blue, low expression; red, high expression), measured in standard deviations from row (genewise) mean. (**F**)—Schematic overview of the major cholesterol homeostatic pathways that may be modulated by HDAC inhibitors. These predictions are based on RNA sequencing data, assuming that corresponding changes occur at the protein levels. Black arrows indicate cholesterol homeostasis under resting conditions (control); orange arrows show potential effects of HDAC inhibitor treatment, with arrow thickness reflecting the direction of change. Some elements used to create this figure are adapted from Bioicons: cell_membrane_semi_circle icon by Helicase 11 undefined is licensed under CC-BY 4.0; receptor icon by Jan-Jezierski is licensed under CC0; lipid-hdl-2 icon by Servier https://smart.servier.com/ is licensed under CC-BY 3.0.

Gene Ontology analysis of differentially expressed genes (DEGs) from the previously generated RNA-seq dataset [43] revealed a significant enrichment of the “sterol biosynthetic process” term (Figure 2A). Heatmap clustering further demonstrated coordinated transcriptional changes across the cholesterol homeostasis network, encompassing genes involved in cholesterol synthesis, transport and uptake, elimination, and efflux (Figure 2A). A number of transcription factors and regulators of cholesterol homeostasis demonstrated differential expression upon HDAC inhibition (Figure 2B). We also observed a pronounced downregulation of genes involved in cholesterol synthesis, transport (apolipoproteins) and cellular uptake (LDL receptors) (Figure 2C,D). Downregulation was accompanied by an upregulation of major genes that control cholesterol efflux (ABC transporters) and metabolic elimination (Figure 2E). Additional protein–protein interaction screening revealed a tightly connected network of the proteins encoded by these genes, with the densest connections among key cholesterol metabolism gene nodes (Supplementary Figure S2).

We hypothesized that these predicted epigenetically triggered transcriptional changes may contribute to the metabolic remodeling by suppressing the anabolic cholesterol programs and activating catabolic and efflux pathways (Figure 2F). To experimentally validate transcriptomic data, we performed a quantitative PCR for a subset of representative genes selected from each functional category. Recognizing that cholesterol homeostatic mechanisms often depend on cellular context, we extended our analysis to compare the TSA-mediated transcriptional responses in neuron-enriched and glia-enriched rat primary cultures maintained under their respective culture-specific conditions.

### 2.2. HDAC Inhibitor Trichostatin A Differentially Regulated Expression of Genes Encoding Cholesterol Synthesis Enzymes in Primary Neuron and Mixed Glial Cultures

Cholesterol is synthesized from acetyl-CoA through multistep enzymatic reactions. The expression of more than 30 genes involved in cholesterol synthesis, uptake and homeostasis is tightly regulated by sterol regulatory element-binding proteins (SREBPs), which bind sterol response elements (SREs) within the regulatory regions of their target genes [45,46,47].

**Figure 3:**
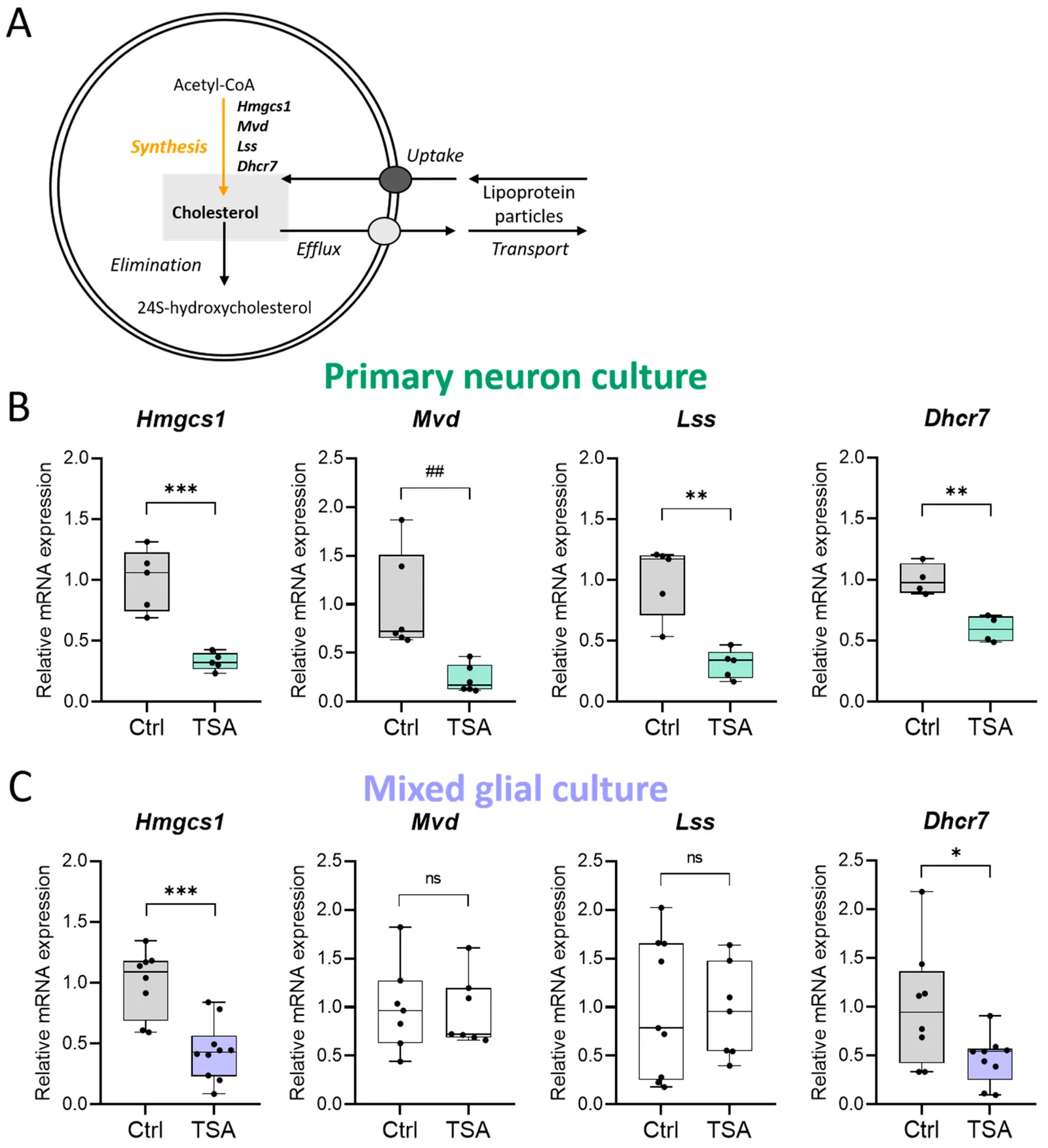
Differential regulation of cholesterol synthesis gene expression by trichostatin A (TSA, 100 nM) in primary neuron and mixed glial cultures. (**A**)—Schematic overview of the major pathways involved in cholesterol homeostasis, with the cholesterol synthesis pathway highlighted in orange. Genes selected for qPCR validation are indicated on the scheme. (**B**,**C**)—qPCR analysis of *Hmgcs1*, *Mvd*, *Lss*, and *Dhcr7* gene expression in control versus TSA-treated primary neuron cultures (**B**), or mixed glial cultures (**C**). Each group consisted of “*n*” individual values derived from “(*n*)” biological replicates, where “*n*” was 4–6 (3–6) for neurons and 7–10 (6–7) for glial cells. Data (fold change) were shown as median ± SD. *—*p* < 0.05, **—*p* < 0.01, ***—*p* < 0.001, unpaired Student’s *t*-test. ^##^—*p* < 0.01, Mann–Whitney U test. ns—not significant. Exact *p*-values, adjusted *p*-values, and the number of replicates are provided in Supplementary Table S1.

Recent studies indicate that various HDAC inhibitors can suppress the expression of SREBP2 target genes involved in cholesterol synthesis and uptake, thereby reducing cellular cholesterol levels in different non-neuronal cell types and in SH-SY5Y human neuroblastoma cells [32,36,42]. Consistent with these observations, transcriptomic analysis of rat primary neuron cultures revealed that prolonged TSA treatment (19 h, 100 nM) significantly decreased the mRNA expression of multiple genes encoding cholesterol biosynthesis enzymes (Figure 2C). We validated these findings by qPCR for selected genes (Figure 3A), including: (1) *Hmgcs1*, encoding 3-Hydroxy-3-Methylglutaryl-CoA Synthase 1, which catalyzes an early step in cholesterol synthesis—the conversion of acetyl-CoA to mevalonate; (2,3) *Mvd* (Mevalonate Diphosphate Decarboxylase) and *Lss* (Lanosterol Synthase), which participate in the production of downstream pathway intermediates; and (4) *Dhcr7*, encoding 7-Dehydrocholesterol Reductase, which catalyzes the final-step of the Kandutsch–Russell pathway of cholesterol biosynthesis (Figure 3B).

In mixed glial cultures, the TSA-mediated effects were more restricted, with significant changes detected only for *Hmgcs1* and *Dhcr7*, both the established SREBP-regulated genes [32,47]. In contrast, the *Mvd* and *Lss* gene expression was not significantly altered (Figure 3C).

This differential sensitivity to HDAC inhibition in different rat primary cultures likely reflects the cell-type specific differences in chromatin accessibility, basal pathway activity, and transcriptional regulation of cholesterol biosynthesis. Thus, neurons may exhibit broader downregulation of stimulus-inducible cholesterogenic genes, likely due to redistribution of the universal substrate acetyl-CoA towards the histone acetylation, whereas glial cells may maintain a more constitutively active cholesterol biosynthesis program and consequently display a more limited transcriptional response.

We also examined the mRNA expression of *Srebf2*, which encodes the master cholesterol-regulatory transcription factor SREBP2, in primary neuron cultures and mixed glial cultures. We detected no statistically significant changes or trends in either culture type (Supplementary Figure S3A,B). This finding is consistent with previous reports indicating that HDAC inhibitors may affect SREBP2 activity post-transcriptionally by reducing the abundance of its proteolytically cleaved, transcriptionally active protein isoform, rather than by altering the *Srebf2* transcription [42]. Such changes have been associated with impaired SREBP2 processing and/or increased protein degradation, accompanied by the downregulation of cholesterol biosynthesis genes and depletion of cellular cholesterol. These observations led us to hypothesize that HDAC inhibitors may similarly regulate the cholesterol-associated gene expression in our experimental models without altering the *Srebf2* mRNA expression. However, this possibility requires further functional validation, including analysis of the precursor and mature SREBP2 protein isoforms, their subcellular localization, and direct measurements of cellular cholesterol levels.

### 2.3. HDAC Inhibitor Trichostatin A Differently Regulated Expression of Genes Encoding Components of Cholesterol Transport and Uptake in Primary Neuron and Mixed Glial Cultures

Astrocytes are a type of glial cell that functions as the major source of cholesterol in the brain. Newly synthesized cholesterol is packaged into apolipoprotein-containing lipoprotein particles, predominantly ApoE-containing particles [22], which mediate intercellular cholesterol transport [48]. These cholesterol-rich particles can bind specific cell-surface receptors, including LDLR, LRP1, and VLDLR, on neurons and other recipient cells, thereby promoting cholesterol uptake through receptor-mediated endocytosis [12]. Following internalization, the cholesterol transport proteins NPC1 and NPC2 (Niemann–Pick C1 and C2), located in the membranes of late endosomes and lysosomes and luminal compartments, facilitate the intracellular trafficking of unesterified cholesterol to the endoplasmic reticulum and plasma membranes [12,49,50].

Compared with the regulation of cholesterol biosynthesis genes, the epigenetic regulation of apolipoprotein, NPC1/2, and lipoprotein receptor genes remains poorly characterized [14]. Available evidence indicates that HDAC inhibitors can alter the mRNA expression of LDLR (↓ [32,36], ↑ [37]) NPC1 (↑ [32]), ApoD (↓ [41]) and ApoE (↑ [32], ↓ [36]), although the direction and magnitude of these effects appear to depend strongly on cellular context.

**Figure 4:**
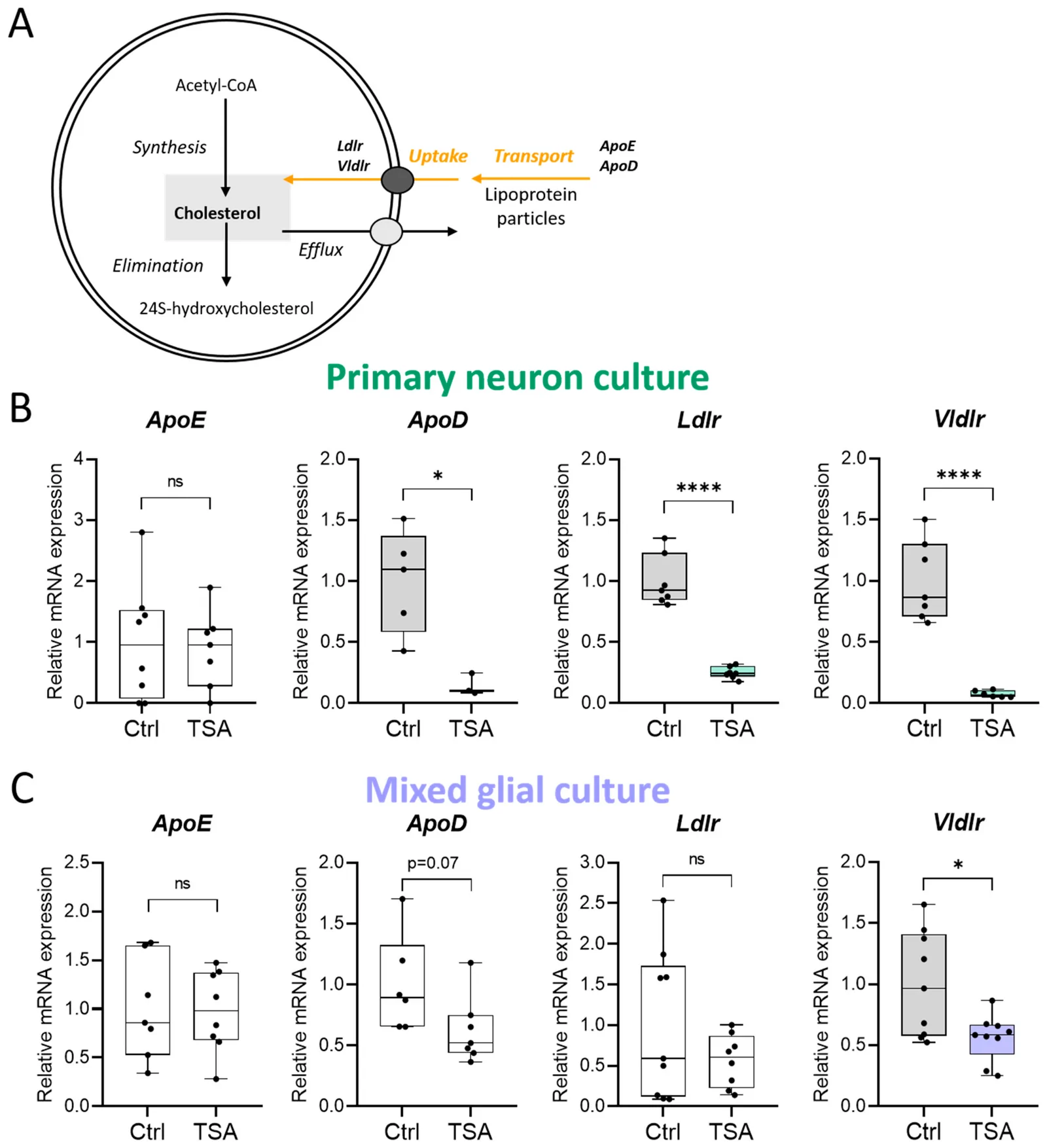
Trichostatin A (TSA, 100 nM) differentially regulates genes involved in cholesterol transport and uptake in primary neuron and mixed glial cultures. (**A**)—Schematic overview of the major cholesterol homeostatic pathways, with components related to cholesterol transport and uptake highlighted in orange. Genes selected for qPCR validation are indicated on the scheme. (**B**,**C**)—Relative mRNA expression of genes encoding apolipoproteins (*Apoe*, *Apod*) and lipoprotein receptors (*Ldlr*, *Vldlr*), measured by qPCR in control versus TSA-treated primary neuron cultures (**B**), or mixed glial cultures (**C**). Each group consisted of “*n*” individual values derived from “(*n*)” biological replicates, where “*n*” was 3–8 (3–7) for neurons and 6–9 (6–8) for glial cells. Data (fold change) were shown as median ± SD. *—*p* < 0.05, ****—*p* < 0.0001, unpaired Student’s *t*-test. ns—not significant. Exact *p*-values, adjusted *p*-values, and the number of replicates are provided in Supplementary Table S1.

In the present study, the transcriptomic analysis revealed the heterogeneous expression patterns among genes involved in intercellular and intracellular cholesterol transport and uptake (Figure 2D). Based on these findings, we selected three DEGs for qPCR validation: (1) *Apod*, encoding apolipoprotein D, which is broadly expressed in glial cells (astrocytes, oligidendrocytes) and neurons and contributes to lipid and cholesterol transport [51,52]; (2,3) *Ldlr* and *Vldlr*, encoding low-density lipoprotein receptors that bind apolipoprotein-containing lipoprotein particles and facilitate cholesterol uptake (Figure 4A). In addition, heatmap analysis suggested a trend toward reduced *Apoe* gene expression. Because ApoE is the predominant apolipoprotein produced by astrocytes in the brain [53], we included the *Apoe* gene in the qPCR analysis.

TSA treatment did not significantly alter the *Apoe* mRNA expression in either primary neuron cultures or mixed glial cultures (Figure 4B,C). However, in neuron-enriched cultures, TSA significantly reduced the mRNA levels of *Apod*, *Ldlr*, and *Vldlr* (Figure 4B), consistent with the transcriptomic data (Figure 2D) and with previous reports [32,36,41]. In mixed glial cultures, TSA significantly decreased the *Vldlr* gene expression, whereas the *Apod* and *Ldlr* expression remained unchanged (Figure 4C).

### 2.4. Trichostatin A Differentially Regulated Expression of Genes Responsible for Cholesterol Elimination and Efflux in Primary Neuron and Mixed Glial Cultures

Cholesterol homeostasis in brain cells is maintained through several complementary pathways. First, excess unesterified cholesterol can be exported from neurons and glial cells to extracellular lipid acceptors, including apolipoproteins, by the specific ATP-binding cassette (ABC) transporters, located at the plasma membrane [25,27,54]. In neurons, this pathway is responsible for approximately 75% of total cholesterol efflux [54]. Second, in cells expressing cholesterol 24-hydroxylase (CYP46A1), which is predominantly expressed in neurons [28], excess cholesterol can be converted into 24S-hydroxycholesterol, a more soluble derivative that readily crosses plasma membranes, exits the brain, and undergoes peripheral elimination.

Several transcription factors, including PPARγ and the liver X receptor isoforms LXRα and LXRβ, regulate gene expression to stimulate cholesterol efflux through ABC transporters [13,27,55,56,57,58] and decrease cholesterol accumulation [13,58,59]. Previous studies suggest that components of cholesterol elimination pathways, including *Cyp46a1*, *Abca1*, and *Abcg1*, can also be regulated epigenetically with corresponding changes in mRNA expression in non-neuronal cells and neuroblastoma cell line [14,32,38,40].

**Figure 5:**
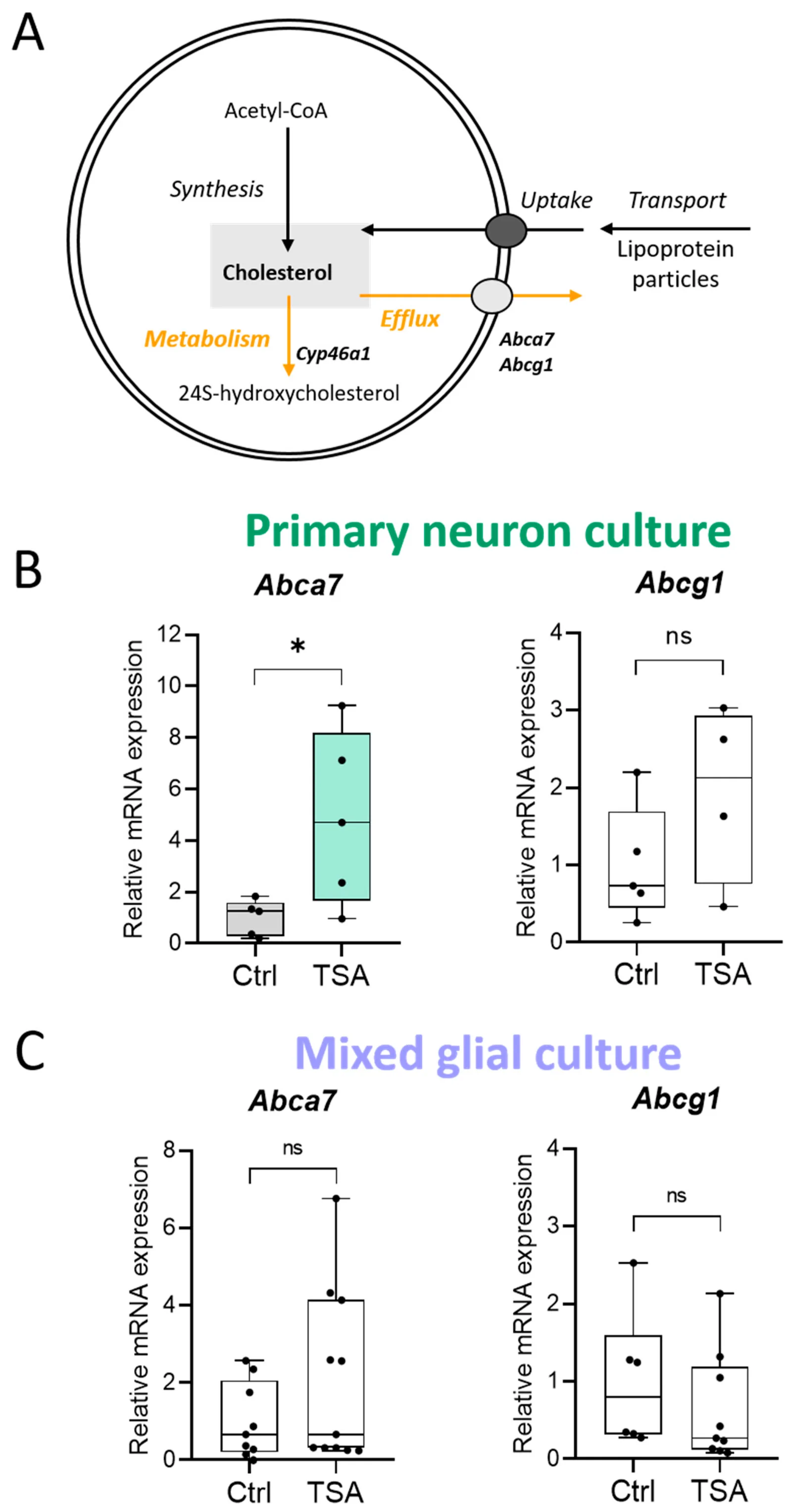
Trichostatin A (TSA, 100 nM) differentially regulates genes involved in cholesterol efflux in primary neuron and mixed glial cultures. (**A**)—Schematic overview of the major cholesterol homeostatic pathways, with components related to cholesterol efflux highlighted in orange. Genes selected for qPCR validation are indicated on the scheme. (**B**,**C**)—Relative mRNA expression of genes encoding ABC transporters (*Abca7*, *Abcg1*) and cholesterol 24-hydroxylase (*Cyp46a1*), measured by qPCR in control versus TSA-treated primary neuron cultures (**B**), or mixed glial cultures (**C**). Each group consisted of “*n*” individual values derived from “(*n*)” biological replicates, where “*n*” was 4–5 (4–5) for neurons and 6–11 (6–10) for glial cells. Data (fold change) were shown as median ± SD. *—*p* < 0.05, unpaired Student’s *t*-test. ns—not significant. Exact *p*-values, adjusted *p*-values, and the number of replicates are provided in Supplementary Table S1.

In the present study, transcriptomic analysis revealed upregulation of *Pparg* (encoding PPARγ) and *Nr1h2* genes (encoding LXRβ) together with differential expression of several downstream target genes involved in cholesterol efflux regulation (Figure 2B,E). We, therefore, selected the representative genes for qPCR validation, including (1) *Abca7*, the closest homolog of *Abca1* [60], which mediates cholesterol efflux that can compensate the ABCA1 deficiency [61], and has been linked to increased Alzheimer’s disease (AD) risk through its role in mitochondrial lipid metabolism regulation [62]; (2) *Abcg1*, one of the key mediators of cholesterol efflux to high-density lipoprotein particles (HDL) [25,54,63]; and (3) *Cyp46a1*, which encodes a neuron-specific enzyme responsible for cholesterol elimination [64] (Figure 5A).

In primary neuron cultures, TSA significantly increased the *Abca7* gene expression and produced a modest, non-significant trend toward increased *Abcg1* expression (Figure 5B). Neither effect was observed in mixed glial cultures (Figure 5C). To determine whether other members of the ABC transporter family were affected under our experimental conditions, we additionally examined *Abcg2*, another differentially expressed gene identified in our transcriptomic dataset (Figure 2E). Although ABCG2, encoded by the *Abcg2* gene, is a multidrug efflux transporter and is not directly implicated in cholesterol efflux from neurons or glia, its activity depends on cellular cholesterol levels [65]. TSA significantly increased the *Abcg2* mRNA expression in both neuronal and mixed glial cultures (Supplementary Figure S4A,B). These findings further support the possibility that epigenetic mechanisms contribute to the broader transcriptional regulation within the ABC transporter family.

For analysis of *Cyp46a1* gene expression, we tested three independent primer pairs. Each produced off-target amplicons, potentially compromising amplification efficiency and the reliability of quantification. Nevertheless, despite these technical limitations, our initial findings suggest that TSA does not influence *Cyp46a1* gene expression under our experimental conditions.

Studies suggest that ABCA1 and ABCG1 act cooperatively to mediate cholesterol efflux: ABCA1 first transfers lipids to lipid-poor extracellular acceptors, such as ApoA-I and ApoE, initiating the formation of nascent HDL particles, whereas ABCG1 promotes further cholesterol efflux to mature HDL [25,54,63]. Given the functional similarity between ABCA7 and ABCA1, it is plausible that ABCA7 may similarly cooperate with ABCG1 to facilitate cholesterol efflux from cultured neurons, where this mode of cholesterol clearance is predominant [54]. However, this hypothesis requires further validation, as the cell-type distribution of ABCA7 remains incompletely defined and, as highlighted in a recent review, may vary across different brain cell types [66].

## 3. Discussion

Multiple studies indicate that cholesterol metabolism in distinct brain cell populations is critically involved in regulation of both physiological and pathological processes in the brain [1,3,4,5,6,18].

Chromatin remodeling may contribute to cholesterol metabolic reprogramming by directly modulating the transcription of cholesterol-related genes. For instance, HDAC9 inhibition can enhance histone acetylation at the promoters of the nuclear ligand-activated receptor gene *Pparg* and its downstream targets *Abca1* and *Abcg1* in macrophages, facilitating their transcription [40]. However, nearly all studies of the epigenetic regulation of cholesterol homeostasis have focused on peripheral cell types or neuron-like cell lines [32,36,37,38,40,41,42], whereas the regulation in brain cells remains largely unexplored [39].

**Figure 6:**
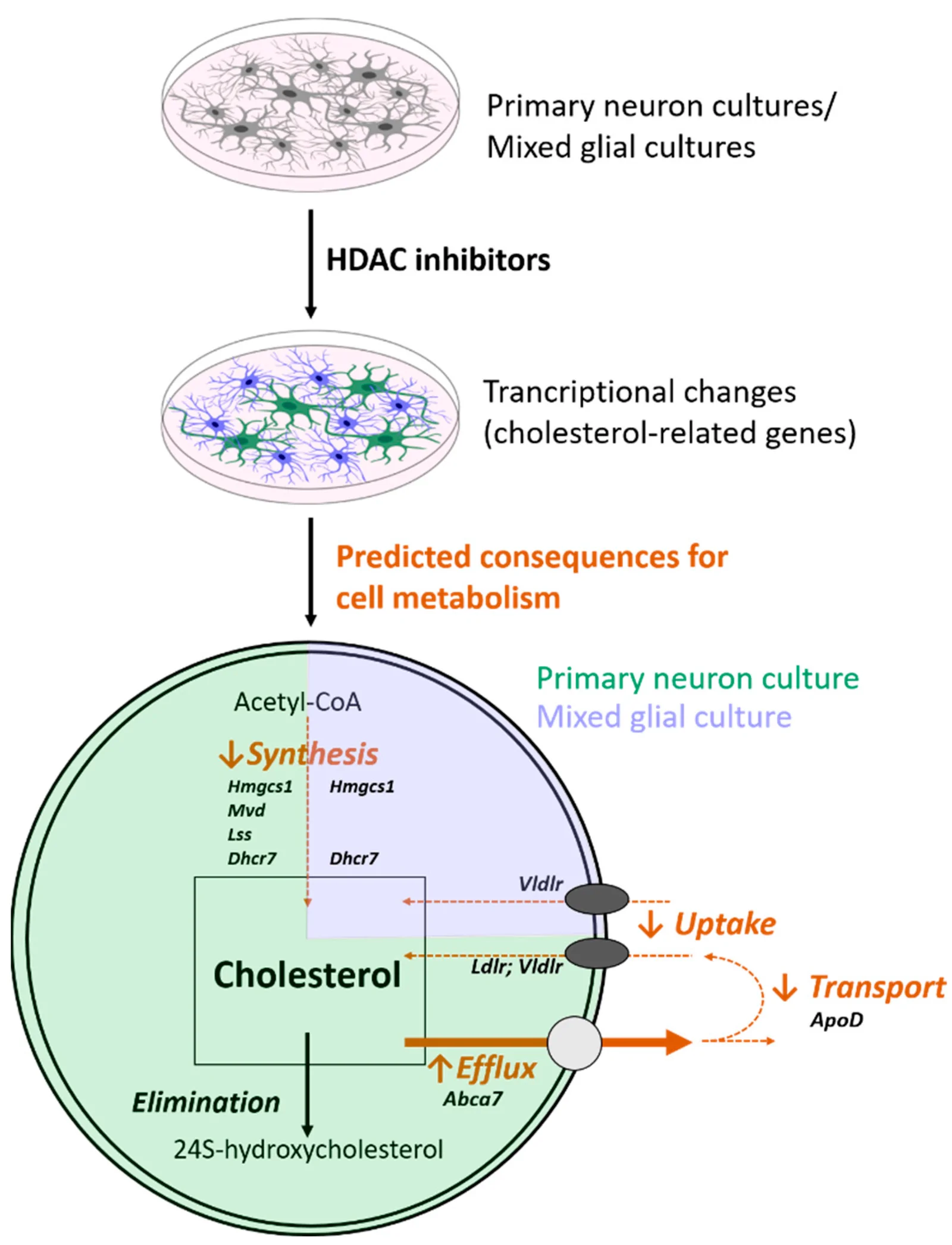
Proposed model of cholesterol homeostasis pathways modulated by HDAC inhibitors in neuron-enriched and glia-enriched rat primary cultures, maintained under culture-specific conditions. Cell cultures were treated with the HDAC inhibitor trichostatin A, which induced the transcriptional changes. The integrated model at the bottom of the scheme depicts a hypothetical cell and summarizes the differentially expressed genes encoding components of cholesterol metabolism that were validated by qPCR (indicated in italics). The potential downstream consequences of these transcriptional changes for cellular metabolism in primary neuron and mixed glial cultures are shown in their respective colors, with the direction of effects highlighted in orange.

To address this gap, we compared the transcriptional responses of neuron-enriched and glia-enriched rat primary cultures, maintained under culture-specific conditions, to the HDAC inhibitior trichostatin A (TSA), focusing on genes involved in cholesterol homeostasis. As described in the Methods section, both culture models contain multiple brain cell populations; therefore, the findings should not be interpreted as responses of purified neurons or individual glial subtypes. Nevertheless, TSA induced distinct transcriptional profiles of cholesterol-related gene families in these two culture models. In primary neuron cultures, TSA produced more extensive transcriptional changes (Figure 2), which, at the protein level, may contribute to reduction in the intracellular cholesterol accumulation (Figure 3B, Figure 4B and Figure 6) and elevation of the cholesterol efflux (Figure 5B and Figure 6). In contrast, the response in mixed glial cultures was less pronounced and, at the protein level, potentially would have led to reduced intracellular cholesterol accumulation (Figure 3C, Figure 4C and Figure 6).

These findings raised two questions: what underlies the differential sensitivity of cell cultures to HDAC inhibition, and how might these responses be regulated? In the following sections we will discuss possible hypotheses that may explain the observed results.

### 3.1. What Underlies the Differential Sensitivity of Cell Cultures to HDAC Inhibition?

Differences in sensitivity of neuron-enriched and glia-enriched primary cultures to HDAC inhibition likely reflect both cell-intrinsic properties and extracellular, culture-dependent factors. Their divergent transcriptional responses may arise from differences in chromatin accessibility and cell-type-specific transcriptional regulation of cholesterol metabolism. Various transcription factors, including members of the LXR, SREBP, and Sp families, interact dynamically with the promoter regions of cholesterol-related genes as a part of multiprotein activator or repressor complexes, regulating their transcription [37,67,68,69]. Re-analysis of our transcriptomic data [44], aligned to the updated Rnor7 genome annotation [43], suggested that TSA treatment of neuron cultures may downregulate expression of the *Srebf2* gene encoding SREBP2, and upregulate the *Pparg* and *Nr1h2*, which encode PPARγ and LXRβ, respectively (Figure 2B). It is widely recognized that SREBPs primarily modulate genes involved in cholesterol biosynthesis and lipoprotein receptor-mediated uptake [45,46,70], whereas the PPARγ/LXRs axis promotes the expression of cholesterol-efflux genes [13,27,55,56,57,58]. These observations are consistent with our working hypothesis regarding the epigenetic regulation of brain cholesterol homeostasis (Figure 2F), and, at the transcription levels, are partially supported by qPCR results (Figure 6). However, direct involvement of these transcription factors in the regulation of cholesterol-related gene networks in our experimental condition was not directly validated.

Culture conditions can also influence basal cholesterol homeostasis. Serum present in the culture medium contains cholesterol, lipoproteins, growth factors, hormones, and other bioactive molecules that can affect sterol availability and the regulation of cholesterol homeostasis. Available data suggest that cholesterol delivered by serum LDL lipoproteins can suppress the processing of the master transcription factor SREBP2 and retain its inactive precursor in extranuclear compartments [71]. Conversely, cultivation of cells under serum-free conditions can promote SREBP2 cleavage and translocation of its active form to the nucleus, where it can activate the downstream cholesterol-related genes. Serum deprivation has also been reported to increase SREBP2 and SREBP1 protein expression [72]. In our experiments, the primary neuron cultures were maintained in serum-free medium with limited access to exogenous cholesterol and lipoproteins, and may, therefore, rely more strongly on endogenous cholesterol synthesis. In contrast, mixed glial cultures were maintained in serum-containing medium and exposed to exogenous lipids and lipoproteins, which may have reduced their dependence on endogenous cholesterol production. Accordingly, even prior to TSA treatment, these two culture models may have differed in basal sterol availability, SREBP2 activity, cholesterol biosynthesis, and, probably, in the expression of cholesterol-related genes. These potential differences represent an important confounding factor and preclude attributing the distinct responses to TSA solely to cell-intrinsic properties.

Overall, the distinct responses to HDAC inhibition may reflect a combination of cell- or culture-type-specific transcriptional regulation and medium-dependent metabolic adaptation. However, the present study cannot determine the relative contribution of these factors, which therefore requires further careful investigation.

### 3.2. Potential SREBP-Mediated Transcriptional Regulation of Cholesterol Homeostasis

Although the direct involvement of master transcription factors in regulating cholesterol-related gene networks was not experimentally validated under our experimental conditions, SREBP2 represents a plausible candidate that may mediate some of the observed transcriptional changes.

SREBPs are central regulators of cholesterol homeostasis that provide a sterol-sensitive negative feedback loop [14]. When intracellular cholesterol levels decline, the proteolytically activated SREBP isoforms translocate to the nucleus and form dynamic nuclear droplets that concentrate the transcription machinery at target genes [73]. There, SREBP binds to sterol-responsive regulatory elements (SREs) in cholesterol-related gene loci (for example, *Hmgcs1*, *Hmgcr*, and *Ldlr*), co-localizing with various gene-specific co-regulators, including Sp1, NF-Y, and CREB [67,74], as well as BRD4, MED1, MED15, and p300 [73], that are required for efficient transcriptional activation [74]. SREBPs can also interact directly with the histone acetyltransferases CBP and p300 [70,75], which can increase chromatin accessibility through histone acetylation and facilitate the expression of target genes. Indeed, binding of SREBP2 and its co-activators to sterol-regulated promoter regions was shown to correlate with the increased histone H3 acetylation [67]. In a related study, H3K27 hyperacetylation at super-enhancers, enriched for SP1, NF-Y, KLF3, SREBP1a, and SREBP2 binding motifs, positively correlated with cholesterol-related gene expression [73]. Conversely, elevated cellular cholesterol reduced the recruitment of these transcription factors to relevant regulatory regions and was accompanied by a decreased histone acetylation [67].

Given this relationship, the HDAC inhibitor-mediated histone hyperacetylation might be expected to enhance the transcription of cholesterol-related genes. Contrary to this prediction, however, both our data and previous reports indicate that HDAC inhibitors can suppress the expression of SREBP2 downstream genes involved in cholesterol synthesis and uptake in various non-neuronal and neuron-like cell types, as well as in neurons [32,36,39,42]. One possible explanation is that the HDAC inhibition alters the acetyl-CoA availability and its distribution among the competing biosynthetic and epigenetic pathways, including cholesterol synthesis and histone acetylation. Recent studies suggest that disruption of epigenetic–metabolic connections, either through microRNA-mediated suppression of cholesterol biosynthesis, or through increased histone acetylation, can alter the cellular acetyl-CoA pool and redirect it towards histone acetylation [30,76]. Such redistribution may promote the transcription of synaptic plasticity-related genes [30], and alter cellular metabolism [76]. In addition, widespread chromatin de-condensation may alter competition among genomic loci for shared transcriptional machinery and pre-assembled “transcription factories” [77]. This redistribution could potentially reduce the recruitment or transcriptional activity of SREBP-regulated loci, and, thereby, limit expression of the corresponding cholesterol-related genes. Although such a mechanism is plausible, it requires direct validation through locus-specific chromatin profiling, analyses of transcription factor occupancy, and assessment of 3D-genome organization.

Another plausible explanation for the HDAC inhibitor-mediated effects is post-transcriptional regulation of cholesterol-related proteins, particularly SREBP2. Acetylation of SREBPs by the histone acetyltransferases CBP and p300 within its DNA binding domain protects the protein from ubiquitin-mediated degradation and enhances cholesterol synthesis [75]. Consistent with this model, recent data indicate that HDAC inhibitors do not necessarily alter the *Srebf2* mRNA expression, but can reduce the abundance of proteolytically cleaved, transcriptionally active SREBP2 isoform, suggesting its impaired processing and/or increased degradation [42]. This decrease was accompanied by downregulation of cholesterogenic genes and depletion of cellular cholesterol. Therefore, changes in SREBP2 protein abundance, processing, stability, or subcellular localization may contribute to the HDAC inhibitor-mediated effects, even when *Srebf2* transcription remains unchanged.

The relative contributions of these mechanisms remain to be determined. Nevertheless, in the present study, TSA elicited transcriptional responses in the same direction in both neuron-enriched and glia-enriched cultures, although the number of affected SREBP2 target genes differed between the two culture models (Figure 6). Primary neuron cultures showed broader downregulation of genes involved in cholesterol synthesis (*Hmgcs1*, *Mvd*, *Lss*, *Dhcr7*), transport (*Apod*), and uptake (*Ldlr*, *Vldlr*) (Figure 3B and Figure 4B). In contrast, the response in mixed glial cultures was more restricted, involving *Hmgcs1*, *Dhcr7*, and *Vldlr* genes (Figure 3C and Figure 4C).

These differences likely reflect, at least in part, the distinct functional roles of neuronal and glial cells in brain cholesterol homeostasis. According to this “division-of-labor” model, neurons have a relatively limited capacity for efficient *de novo* cholesterol synthesis [19] and prioritize resources for activity-dependent plasticity, which requires extensive chromatin remodeling and transcription of plasticity-related genes (reviewed in [78]). In contrast, glia serve as the principal source of cholesterol, supporting neuronal function [19]. High cholesterol demand makes neurons highly vulnerable to intracellular cholesterol dysregulation, so they possess specialized protective mechanisms to prevent the cholesterol overload, such as CYP46a1-mediated cholesterol elimination [64]. Consequently, the neuronal cholesterol-related genes may be more sensitive to perturbations in chromatin regulation, which can explain the extensive transcriptional response to TSA (Figure 6). By contrast, glial cells maintain more constitutive cholesterol synthesis, and have a greater capacity to redistribute excess cholesterol, whether through internal storage or export to neurons. This metabolic flexibility likely explains their weaker transcriptional response to HDAC inhibition (Figure 6). Consequently, perturbing cholesterol metabolism with TSA may further exacerbate these cell-specific differences by engaging various downstream regulatory pathways.

### 3.3. Potential LXR-Mediated Transcriptional Regulation of Cholesterol Homeostasis

Although the direct involvement of master transcription factors in regulating cholesterol-related gene networks was not experimentally validated under our experimental conditions, members of the LXR family represent plausible candidates that may mediate some of the observed transcriptional changes.

Acting as both transcriptional activators and repressors, master-transcription factor LXRs can exert different effects on the genes related to cholesterol metabolism (*Abca1*, *Srebf1*) in tissue-specific manners [68]. In the absence of oxysterols (the endogenous LXR ligands generated during cholesterol metabolic conversion), LXRs recruit HDACs-containing co-repressor complexes NCoR or SMRT to the promoter regions of target genes to repress transcription [68]. In contrast, agonist binding or genetic deletion of the LXRs promotes co-repressors dissociation from the promoter regions, which facilitates histone acetylation and may potentiate transcription [68].

Given the high sequence homology [60] and functional similarity between the *Abca1* and *Abca7* [61], it was initially reasonable to hypothesize that these transporters might be regulated by similar mechanisms. This model could account for the selective TSA-mediated increase in the *Abca7* mRNA expression observed in neuron cultures (Figure 5B), as well as the predicted upregulation of other ABC transporter genes (*Abca6-9*, *Abcg1*, *Abcg2*) (Figure 2E). However, unlike *Abca1*, which is an established direct LXRs target [68,79], *Abca7* transcriptional regulation appears to be strongly dependent on cellular context and is not consistently associated with LXRs activation [80]. Indeed, evidence from fibroblasts indicates that *Abca7* can be positively regulated by SREBP2, which binds to the SRE sites in the *Abca7* promoter region [56]. An intriguing observation highlights the cell-type specific nature of *Abca7* transcriptional control: cholesterol depletion increases *Abca7* mRNA expression in fibroblasts [56], decreases it in human microglia and astrocytes [80], and has little or no effect in human neuroblastoma cells [80]. These divergent responses emphasize a critical gap in our understanding of the *Abca7* transcriptional control, which requires further examination of cell-type specific regulators, including the potential role of LXRs. Finally, the additional experimental validation of TSA-mediated *Abcg2* upregulation (Supplementary Figure S4) further supports the possibility that epigenetic mechanisms contribute to the broader transcriptional regulation of the ABC transporter family.

From this perspective, the HDAC-mediated regulation of cholesterol efflux genes observed in neurons, but not in glia, may reflect the neuron-specific recruitment or activation of regulatory complexes controlling the transcription of genes related to cholesterol metabolism. Nevertheless, the present data do not establish direct involvement of LXRs, which requires further careful examination.

## 4. Materials and Methods

### 4.1. Animals

The experiments were carried out in newborn (P0-P2) Wistar rats (Pushchino Breeding Facility, Russia). All experimental procedures were conducted in accordance with the European Communities Council Directive of 24 November 1986 (86/609/EEC) on the protection of animals used for scientific purposes. The research protocol was approved by the Ethics Committee of the Institute of higher nervous activity and neurophysiology of the Russian Academy of Sciences (ethical approval N8, 28 October 2024). Experiments were carried out using equipment of the Research Resource Center of IHNA and NPh RAS for functional brain mapping.

### 4.2. Rat Primary Cortical Cultures

Primary cultures were obtained from the neocortex of newborn Wistar rats (P0-P1). Wistar rat pups were anesthetized by hypothermia [81], then decapitated, and the brains were rapidly collected and placed in cold, freshly prepared dissection buffer DMEM (Paneco, Moscow, Russia) supplemented with 17.2 mM HEPES. The brain tissue was minced into small pieces and enzymatically dissociated in trypsin-containing dissection buffer (0.83 mg/mL; MP Biomedicals, Solon, OH, USA) for 15 min at 37 °C in a water bath. To stop digestion, the tissue was centrifuged (2000 rpm, 2 min), washed with cold trypsin-free dissection buffer, and centrifuged again under the same conditions. The cell pellet was mechanically dissociated by pipetting in culture-maintenance medium. Primary cortical neuron cultures were processed and maintained in Neurobasal medium (Gibco, Paisley, UK) supplemented with 1X B-27 (Gibco, Grand Island, NY, USA) and 2 mM alanyl-glutamine (Paneco, Moscow, Russia); mixed glial cultures were processed and maintained in DMEM/F12 (Paneco, Moscow, Russia) supplemented with 10% FBS (Gibco, Paisley, UK), 1X non-essential amino acids (Paneco, Moscow, Russia), 2 mM L-glutamine (Paneco, Moscow, Russia), and 1 mM sodium pyruvate (Paneco, Moscow, Russia). Following dissociation, cells were counted and seeded into 4-well plates coated with poly-D-lysine hydrobromide- (Sigma-Aldrich, Saint Louis, MO, USA) at a density of 3 × 10^5^ cells per well in 0.5 mL culture maintenance medium. One hour post-seeding, the medium was replaced with 1 mL of fresh medium. Cultures were maintained at 37 °C and 5% CO_2_ for two weeks. From day 7 *in vitro* (DIV) onward, the culture medium was partially refreshed every 2–3 days.

On the 14 DIV, HDAC inhibitor trichostatin A (TSA, 100 nM, Sigma-Aldrich, Saint Louis, MO, Sigma-Aldrich, USA) was applied without washout. Control and experimental cultures were grown in wells of the same plate and processed in parallel to minimize intergroup variability. Cultures from at least four biological replicates were used to reduce intragroup variability. All samples were coded for blinded processing and analysis.

The composition of the primary neuron cultures was characterized in our previous study using immunocytochemistry [44], which confirmed that they were not purely neuronal and contained multiple major brain cell populations, including DCX^+^ and NeuN^+^ neurons representing immature and mature neuronal populations, respectively; Olig2^+^ oligodendrocytes and oligodendrocyte precursor cells; GFAP^+^ [44] and S100β^+^ astrocytes (unpublished). Therefore, although these cultures were referred to as neuron-enriched, they contain multiple brain cell populations.

The composition of the mixed glial cultures was not experimentally characterized in our studies. However, previous studies indicate that these cultures contain multiple glial cell types in varying proportions, with astrocytes representing the predominant population (approximately 55–76% of cells), followed by microglia (approximately 21%) and oligodendrocytes (approximately 14%) [82].

### 4.3. Transcriptomic Analysis

Transcriptomic analysis was performed as described previously [43]. For the present study, the differentially expressed gene (DEG) dataset was filtered using more stringent criteria: an adjusted *p*-value < 0.05 and an absolute log_2_ fold change for gene expression values ≥ 1. Gene ontology (GO) enrichment analysis of the DEG dataset was conducted using the clusterProfiler R package, version 4.14.3 [83]. Data visualization and heatmap generation were performed using the ComplexHeatmap R package, version 2.22.0 [84]. Protein–protein interaction network was constructed using the STRING database, v12 (https://string-db.org/).

### 4.4. RNA Extraction and cDNA Synthesis

Total RNA was isolated using the QIAzol Lysis Reagent (Qiagen, Germantown, MD, USA) according to the manufacturer’s protocol. For each sample, an equal amount of total RNA (approximately 800 ng) was treated with DNase I (Thermo Scientific, Waltham, MA, USA) to eliminate potential genomic DNA contamination. Following DNase treatment, first-strand cDNA synthesis was performed using a commercial MMLV reverse transcription kit (Evrogen, Moscow, Russia) with a mixture of random decamer and oligo-dT primers (Evrogen, Moscow, Russia), according to the manufacturer’s protocol. The synthesized cDNA was stored at −20 °C until further qPCR analysis.

### 4.5. qPCR Analysis

Primer pairs were designed to target the coding DNA sequence of the genes of interest, and their amplification efficiency was assessed by qPCR. Reactions were performed in triplicate for each sample in 384-well plates using SYBR-Green master mix (qPCRmix-HS SYBR + LowROX, Evrogen, Moscow, Russia) with the specific primer pairs (Table 1), on a CFX384 Touch Real-Time PCR Detection System (Bio-Rad, Hercules, CA, USA). The thermal cycling protocol included initial denaturation at 95 °C for 5 min, followed by 42 cycles of 95 °C for 30 s, annealing at the primer-specific temperature for 30 s, and elongation at 72 °C for 30 s. Data were analyzed using CFX Manager™ software (v. 3.1; Bio-Rad, Hercules, CA, USA). Target gene expression was normalized to the rat *Hprt* housekeeping gene (Table 1), and relative mRNA expression levels were calculated using the ΔΔCt method and normalized to control values (fold change).

### 4.6. Statistical Analysis

All qPCR experiments were performed in at least four biological replicates. Each group consisted of “*n*” individual values derived from “(*n*)” biological replicates, where “*n*” was 4–8 (3–7) for neuronal cultures and 6–11 (6–10) for glial cultures. The exact number of replicates are listed in Supplementary Table S1.

qPCR data were presented as box-and-whisker plots showing the median, interquartile range (IQR), minimum/maximum values (±SD), and individual data points. Outliers were identified using the 1.5 × IQR criterion and excluded from subsequent analyses. The exact number of outliers are listed in Supplementary Table S1.

Normality of the data distributions was evaluated using the Shapiro–Wilk test. Statistical significance of the differences between the groups was determined using the unpaired Student’s *t*-test for normally distributed data, and the Mann–Whitney U test for non-normally distributed data. Statistical significance was defined as *p* < 0.05. Significance levels were indicated directly in the figures using asterisks or hash signs, depending on the statistical test applied, with their exact thresholds defined in the respective figure captions. The Benjamini–Hochberg correction was applied to the exact *p*-values to account for multiple comparisons. Exact *p*-values, adjusted *p*-values (*p*.adj), and genes meeting the significance thresholds (*p* < 0.05; *p*.adj < 0.05) are listed in Supplementary Table S1.

## 5. Concluding Remarks

Cholesterol homeostasis is an important component of neuroglial communication. Alterations in cholesterol synthesis, transport, efflux, intracellular trafficking, and oxysterol metabolism have been reported in numerous neurodegenerative diseases, including Alzheimer’s, Parkinson’s, and Huntington’s diseases, as well as other neurological disorders [1,3,6,18]. Accordingly, strategies focused on restoring or reprogramming dysregulated cholesterol metabolic pathways may be relevant to the treatment of neuropathologies, associated with metabolic dysfunction.

In the present study, we directly compared the transcriptional responses of neuron-enriched and glia-enriched cultures to the HDAC inhibitor treatment. We observed culture-specific regulation of cholesterol-related genes, which may reflect the complementary roles of neurons and glial cells in maintaining brain cholesterol homeostasis. Coordinated changes in the expression of genes from related families and functional groups may indicate that the observed transcriptional rearrangements are guided by the specific master transcription factors, which, we propose, may include members of the SREBP and LXR families. However, these hypotheses require functional validation. Similar regulation of genes in neuron-enriched and glia-enriched cultures is likely controlled by shared regulatory pathways. In contrast, the differential regulation of most genes in neuronal cultures may involve cell-type-specific transcriptional partners and co-regulators, differences in chromatin accessibility, and integration with other cell-specific signaling pathways. The mechanisms underlying this cell- or culture-type specificity require further investigation. An important unresolved question is whether these transcriptional changes have functional consequences for cholesterol metabolic pathways in our experimental model, and how such changes may influence the cell function. Future studies will be required to address these questions.

Using the same experimental paradigm, we previously showed that HDAC inhibition in neuron-enriched cultures can alter the expression of brain cell-type-specific markers, likely attributed to the early stages of cellular reprogramming process [44]. In a subsequent study, we identified changes in the alternative splicing of plasticity-related genes that correlated with increased neural network activity [43]. Here, we extend these findings by demonstrating that HDAC inhibition induces coordinated, culture-specific changes in the cholesterol-associated gene networks. These transcriptional changes may hypothetically contribute to metabolic remodeling in brain cells, essential for cell differentiation and plasticity-related processes. Taken together, our data reveal an extensive transcriptional reorganization of neuroglial networks, which, in our opinion, may support brain cell differentiation and functional maturation by promoting metabolic remodeling.

## 6. Limitations of the Study

- The HDAC inhibitors have broad and relatively non-selective activities and may induce genome-wide changes in chromatin accessibility and transcription beyond the cholesterol-related genes. Therefore, the observed changes in cholesterol-related gene expression cannot be interpreted as isolated, pathway-specific effects; instead they may reflect broader remodeling of neuronal and glial transcriptional programs. The contribution of indirect transcriptional regulation and cell-type-specific changes across the broader gene set remains to be determined.
- Our conclusions are based on the RNA sequencing and qPCR data and are, therefore, limited primarily to the mRNA abundance. Changes in transcript levels may not accurately predict enzyme activity, protein abundance, or overall pathway capacity. Because the present study did not include direct validation of cholesterol levels, we can only hypothesize that the observed transcriptional changes may contribute to remodeling of cholesterol homeostasis. This possibility requires further validation using functional cholesterol assays.
- Primary neuron cultures and mixed glial cultures were maintained in media with different compositions. These differences may affect basal cholesterol metabolism and the expression of cholesterol-related genes, potentially influencing the response to HDAC inhibition. Accordingly, the results should be interpreted as a comparison between neuron-enriched and glia-enriched cultures maintained under their respective experimental conditions, rather than as a fully controlled comparison of isolated neuronal and glial cell types.
- The mixed glial cultures contained multiple glial cell populations. Consequently, our interpretations centered predominantly on astrocytic cholesterol metabolism may not fully reflect the overall response of glial cells to the TSA treatment.
- The TSA-mediated regulation of cholesterol elimination genes, including *Cyp46a1*, could not be reliably assessed under our experimental conditions. All three primer pairs tested produced off-target amplicons, precluding accurate quantification of the *Cyp46a1* mRNA expression and leaving the effect of TSA unresolved.

## Figures and Tables

**Table 1:** The list of specific primer pairs used for qPCR analysis of cholesterol-related gene expression.

Gene	Function	Primers
*Hmgcs1*	Cholesterol synthesis	for, 5′-TGATCTTTCACTCGCCATACTG-3′; rev, 5′-TCTGGTTGAATAGCTCAGCAC-3′;
*Mvd*		for, 5′-CTGCTGCGAATGGAGACAA-3′; rev, 5′-CTGAGTGGCAATGATGTACTGA-3′
*Lss*		for, 5′-GGCACCAATGGATCACAGAC-3′; rev, 5′-TGGCGATAATACTTCTGGTAGTCA-3′
*Dhcr7*		for, 5′-GAAGACCATTGACATCTGCCA-3′; rev, 5′-TCGGAAGATATAGTAACCCACCA-3′
*ApoE*	Cholesterol transfer	for, 5′-GGAACTGACGGTACTGATGGA-3′; rev, 5′-AGCATGGTGTTTACCTCGTTG-3′
*ApoD*		for, 5′-ACTGCATTCAAGCCAACTACTC-3′; rev, 5′-GGCATCAACGAGAAGAACTGG-3′
*Ldlr*	Cholesterol uptake	for, 5′-GGCAGTAGTGAGTGTATCCATC-3′; rev, 5′-GGCTACCGTGAATACAGGAG-3′
*Vldlr*		for, 5′-TGCCAATAAATTCACTGGGTCA-3′; rev, 5′-GATATTCACATCCTCCATTCTCCA-3′
*Abca7*	Cholesterol efflux	for, 5′-GTGGAGGAAGAAAGGATACAAGAC-3′; rev, 5′-CATGATGTAGCACGAGACCTTC-3′
*Abcg1*		for, 5′-CACAGCATCACTACACTCAACC-3′; rev, 5′-TGCGTTCTTCTATGAGGACTAGG-3′
*Abcg2*		for, 5′-GTGTTAGTACCGATGTCACAGAG-3′; rev, 5′-TCCCAGAATAGCATTAAGACCAG-3′
*Cyp46a1*	Cholesterol elimination	for1, 5′-GCTAAGAAGTATGGTCCTGTTGTG-3′; rev1, 5′-GAATCGCACGGTACATCTTGG-3′
		for2, 5′-GAAGTATGGTCCTGTTGTGAGAG-3′; rev2, 5′-GCTTATACCAGCGCCCATAG-3′
		for3, 5′-GTGCCACCATCGACATCCTG-3′; rev3, 5′-GGTGTTACGGGACGCACTGATAC-3′
*Srebf2*	TF	for, 5′-GGTGTGATTGTCTTGAGCGT-3′; rev, 5′-GATAAGCAGGTCTGTAGGTTGG-3′
*Hprt*	Housekeeping gene	for, 5′-CGTCGTGATTAGTGATGATGAAC-3′; rev, 5′-CAAGTCTTTCAGTCCTGTCCATAA-3′

## Data Availability

Original data are included in the article. Further inquiries can be directed to the corresponding author. The RNA sequencing datasets are available at the Gene Expression Omnibus GEO database with ID GSE297880.

## Supplementary Materials

The following supporting information can be downloaded at: https://www.mdpi.com/article/10.3390/ijms27209231/s1.

## Abbreviations

3D-genome	Three-dimensional genome
ABC	ATP-binding cassette (including ABCA1, ABCA7, ABCG1, ABCG2)
AD	Alzheimer’s disease
ApoA-I	Apolipoprotein A-I
APOD	Apolipoprotein D
APOE	Apolipoprotein E
BRD4	Bromodomain-containing protein 4
CBP	CREB-binding protein
CREB	cAMP-responsive element-binding protein
CYP46A1	Cytochrome P450 family 46 subfamily A member 1 (cholesterol 24-hydroxylase)
DEGs	Differentially expressed genes
DHCR7	7-dehydrocholesterol reductase
GO	Gene Ontology analysis
H3	Histone H3 (including H3K27 residue)
HDAC	Histone deacetylase
HDL	High-density lipoprotein
HMGCS1	3-hydroxy-3-methylglutaryl-CoA synthase 1
KLF3	Kruppel-like factor 3
LDL	Low-density lipoprotein
LDLR	Low-density lipoprotein receptor
LP	Lipoprotein particles
LRP1	Low-density lipoprotein receptor-related protein 1
LSS	Lanosterol synthase
LXR	Liver X receptor
MED1	Mediator complex subunit 1
MED15	Mediator complex subunit 15
miRNA	Micro ribonucleic acid
mRNA	Messenger ribonucleic acid
MVD	Mevalonate diphosphate decarboxylase
NaB	Sodium butyrate
NF-Y	Nuclear transcription factor Y
NPC1/2	Niemann–Pick disease type C1 and C2
Nr1h2	Nuclear receptor subfamily 1 group H member 2 (LXRβ gene)
p300	Histone acetyltransferase p300 (EP300)
Pparg	Peroxisome proliferator-activated receptor gamma (gene)
PPARγ	Peroxisome proliferator-activated receptor gamma (protein)
qPCR	Quantitative (real-time) polymerase chain reaction
RNA-seq	RNA (ribonucleic acid) sequencing
Rnor7	Rattus norvegicus genome assembly 7
Sp	Specificity protein
Srebf2	Sterol regulatory element-binding transcription factor 2 (gene)
SREBPs	Sterol regulatory element-binding proteins (including isoforms 1a and 2)
SREs	Sterol response elements
TF	transcription factors
TSA	Trichostatin A
VLDLR	Very-low-density lipoprotein receptor
