# Supplementary material for: Differential Regulation of Cholesterol-Related Gene Expression by HDAC Inhibition in Neuron-Enriched and Glia-Enriched Rat Primary Cultures
Source: Int J Mol Sci. 2026 Oct 17;27(20):9231. doi: 10.3390/ijms27209231 (PMC13705406; doi:10.3390/ijms27209231)
Supplement: Supplementary file 1 [file ijms-27-09231-s001.zip › ijms-4552231-supplementary.pdf]

Differential Regulation of Cholesterol-Related Gene Expression by HDAC Inhibition in Neuron-Enriched and Glia-Enriched Rat Primary Cultures

Alevtina A. Samorodova , Alexander P. Beletskiy, Pavel M. Balaban and Anastasia A. Borodinova *

Laboratory of Cellular Neurobiology of Learning, Institute of Higher Nervous Activity and Neurophysiology, Russian Academy of Sciences, Moscow 117485, Russia.

* Correspondence: borodinova.msu@mail.ru

Supplementary Table S1. Summary of exact *p*-values, adjusted *p*-values (*p.adj*), genes that passed the significance threshold, and the number of replicates for each experiment.

		Primary neuron cultures				Mixed glial cultures			
Category	Genes	Ctrl	TSA	*p-value*	*p.adj.*	Ctrl	TSA	*p-value*	*p.adj.*
Synthesis	***Hmgcs1***	5 (4); outliers=0	5 (5); outliers=0	0.0005***	0.0024	8 (6); outliers=1	10 (7); outliers=1	0.0003***	0.0018
	Mvd	6 (6); outliers=0	6 (6); outliers=0	0.0022##	0.0066	7 (6); outliers=1	7 (7); outliers=2	0.8429	0.9195
	Lss	5 (5); outliers=1	5 (5); outliers=1	0.0012**	0.0041	9 (6); outliers=0	7 (7); outliers=0	0.8852	0.9237
	Dhcr7	4 (3); outliers=1	4 (4); outliers=1	0.0029**	0.0077	8 (6); outliers=1	9 (7); outliers=2	0.0295*	0.0590
Transport and uptake	***ApoE***	8 (7); outliers=1	7 (7); outliers=2	0.7926	0.9058	7 (7); outliers=1	8 (8); outliers=1	0.9306	0.9306
	ApoD	5 (4); outliers=0	3 (3); outliers=0	0.0155*	0.0338	6 (6); outliers=0	7 (6); outliers=1	0.0717	0.1229
	Ldlr	7 (7); outliers=0	7 (7); outliers=0	<0.0001****	0.0008	9 (6); outliers=0	8 (7); outliers=1	0.2200	0.2984
	Vldlr	7 (7); outliers=0	6 (6); outliers=1	<0.0001****	0.0008	9 (6); outliers=0	9 (7); outliers=2	0.0134*	0.0322
Efflux	***Abca7***	5 (4); outliers=1	5 (5); outliers=1	0.0363*	0.0670	9 (9); outliers=0	11 (10); outliers=	0.4119	0.5203
	Abcg1	5 (4); outliers=2	4 (4); outliers=2	0.1800	0.2880	6 (6); outliers=2	9 (8); outliers=0	0.2238	0.2984
	Abcg2	6 (5); outliers=1	7 (7); outliers=0	<0.0001****	0.0008	6 (6); outliers=1	8 (8); outliers=1	0.0011**	0.0041
TF	***Srebf2***	4 (3); outliers=2	5 (5); outliers=1	0.6591	0.7909	8 (7); outliers=1	11 (9); outliers=2	0.2014	0.2984

Each group consists of "*n*" individual values derived from "(*n*)" biological replicates.
The "*p*-value" column reports exact *p*-values; asterisks indicate statistically significant differences between control and TSA-treated cultures. * – p <0.05, ** – p <0.01, *** – p <0.001, **** – p <0.0001, unpaired Student's t-test. ## – p <0.01, Mann-Whitney U test.
The "*p.adj.*" column shows *p*-values adjusted for multiple comparisons using the Benjamini-Hochberg correction.
Statistically significant values are highlighted in red.
TF – transcription factors.

Western blotting

To validate the effect of trichostatin A (TSA) treatment on histone acetylation, total proteins were extracted from control and TSA-treated rat primary cortical neuron cultures using RIPA buffer supplemented with a protease inhibitor cocktail, according to the standard protocol. For Western blotting, protein samples were mixed with 4x Laemmli sample buffer supplemented with 1 mM dithiothreitol (DTT) and separated by 15% SDS-PAGE (30 mA, 125 V, 80 min). Proteins were then transferred to a nitrocellulose membrane (300 mA, 125 V, 50 min). Protein loading was verified by Ponceau S staining. After blocking, the membrane was incubated overnight at 4 °C with the primary antibodies against histone H3 acetylated at lysine 9 (H3K9ac; 1:750; Cat. #9649, Cell Signaling Technology, Danvers, MA, USA) diluted in blocking buffer (Cat. #12010020, Bio-Rad, Hercules, CA, USA). The next day, the membrane was incubated with horseradish peroxidase (HRP)-conjugated anti-rabbit secondary antibodies (1:2000; Cat. #1706515, Bio-Rad, Hercules, CA, USA) for 1 h at room temperature. Immunoreactive bands were detected using Clarity Western ECL Substrate (Cat. #170-5061, Bio-Rad, Hercules, CA, USA) and visualized with a ChemiDoc Imaging System (Bio-Rad, Hercules, CA, USA). A molecular weight marker (Cat. #26619, Thermo Fisher Scientific, Vilnius, Lithuania) was used as a reference for protein molecular mass.

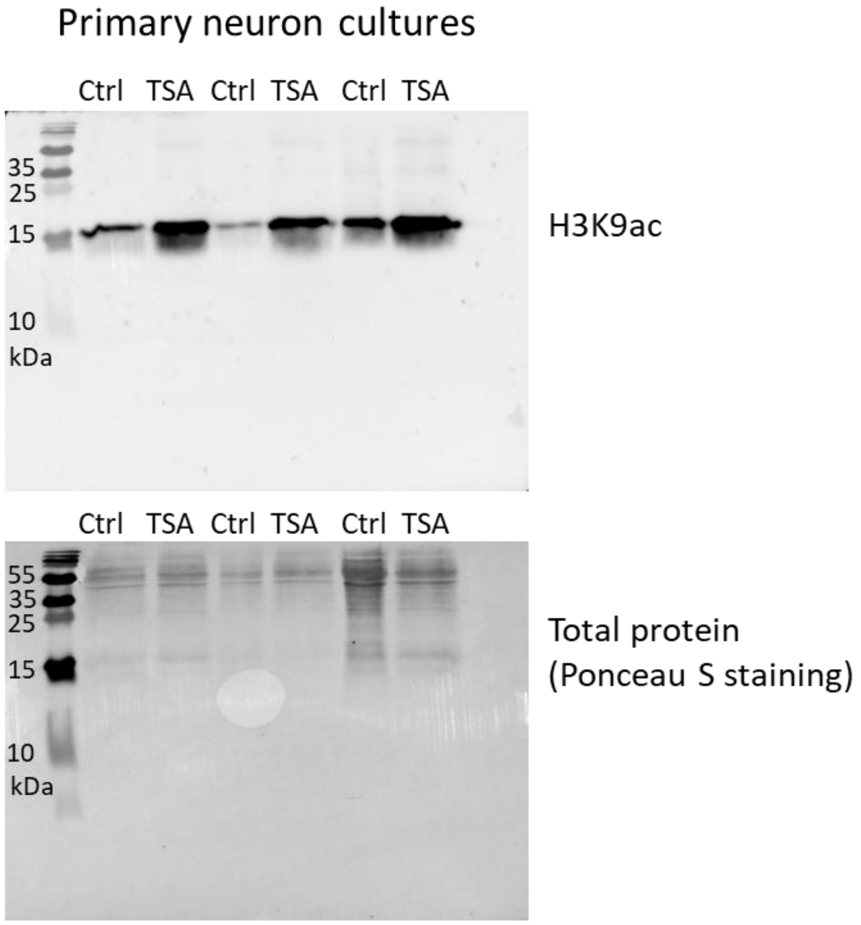

Supplementary Figure S1. Validation of TSA-induced histone hyperacetylation. Western blot analysis of histone H3 acetylated at lysine 9 (H3K9ac; upper panel). Samples were obtained from control (Ctrl) and TSA-treated (100 nM, 19 h) primary cortical neuron cultures (*n* = 3 per group). Total protein loading was verified by Ponceau S staining (lower panel). Molecular-weight markers are indicated in kDa.

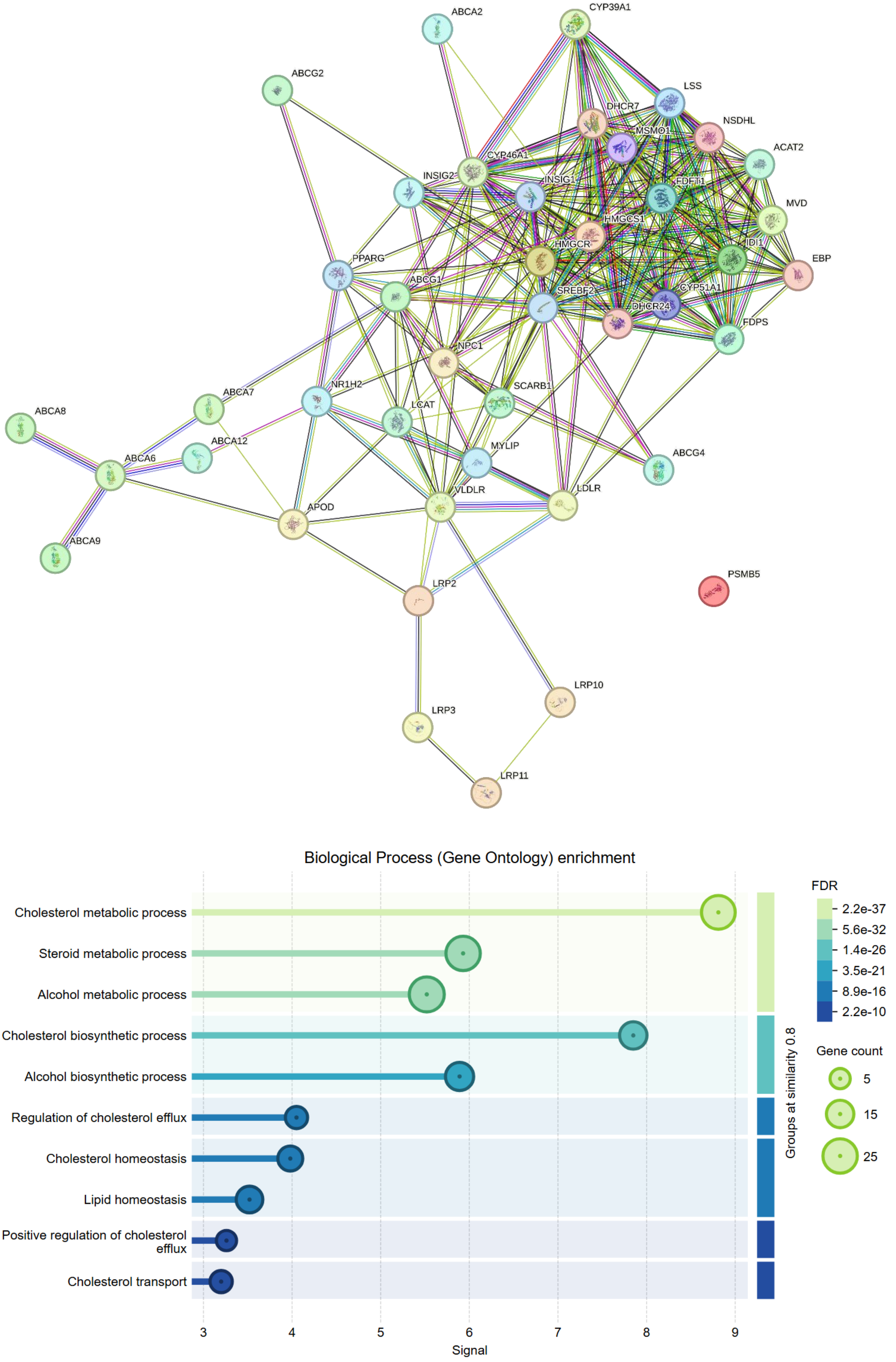

Supplementary Figure S2. Protein-protein interaction screening revealed a tightly connected network among the proteins encoded by the differentially expressed cholesterol-related genes

(from Fig.2B-E). The Gene Ontology (GO) category enrichment table and the corresponding figure generated using the STRING database (string-db.org) illustrate the key biological processes and signaling pathways enriched among the cholesterol metabolism-related genes included in our analysis.

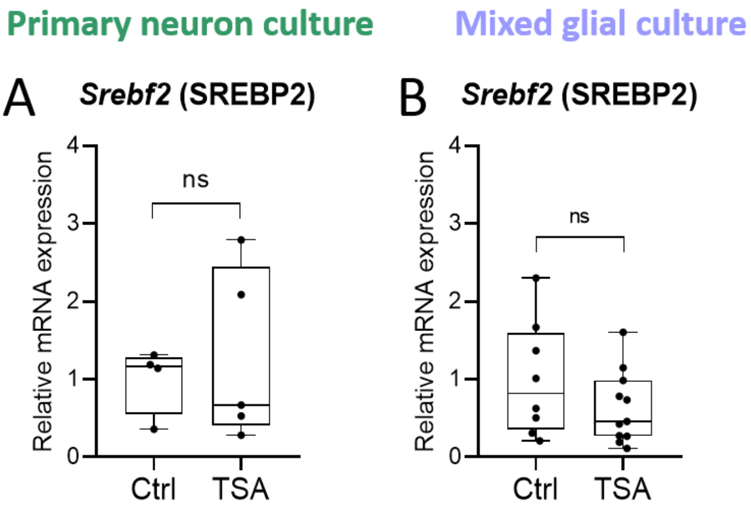

Supplementary Figure S3. HDAC inhibition with trichostatin A (TSA, 100 nM) did not affect the mRNA expression of master transcription factor SREBP2 regulating gene networks associated with cholesterol metabolism. A, B – qPCR analysis of *Srebf2* gene expression in control versus TSA-treated primary neuron cultures (A), or mixed glial cultures (B). Each group consisted of "*n*" values derived from "(*n*)" biological replicates, where "*n*" was 4-5 (3-5) for neurons and 8-11 (7-9) for glial cells. Data (fold change) were shown as median ± SD. ns – not significant. Exact *p*-values, adjusted *p*-values, and the number of replicates are provided in Supplementary Table S1.

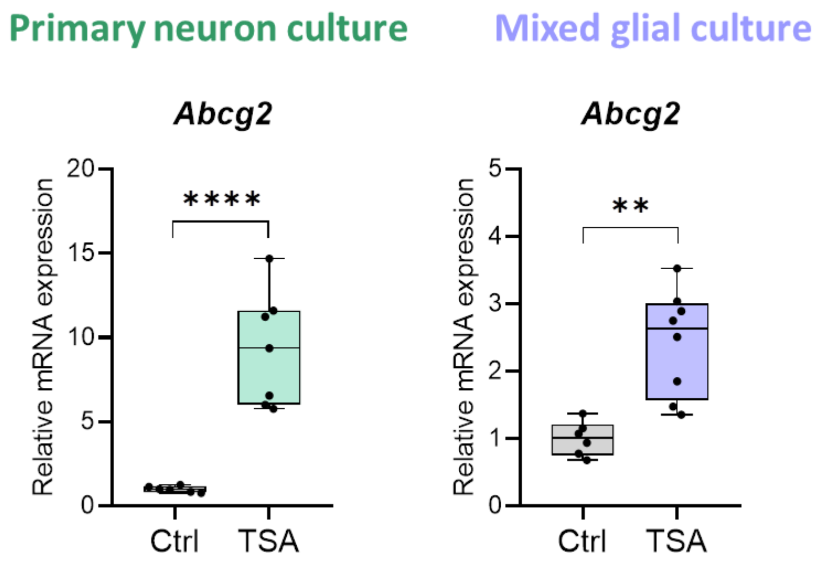

Supplementary Figure S4. HDAC inhibition with trichostatin A (TSA, 100 nM) increases mRNA expression of the *Abcg2* gene from the ABC-transporter's family. A, B – qPCR analysis of the *Abcg2* gene expression in control versus TSA-treated primary neuron cultures (A), or mixed glial cultures (B). Each group consisted of "*n*" values derived from "(*n*)" biological replicates, where "*n*" was 6-7 (5-7) for neurons and 6-8 (6-8) for glial cells. Data (fold change) were shown as median ± SD. ** – $p < 0.01$, **** – $p < 0.0001$, unpaired Student's t-test. Exact *p*-values, adjusted *p*-values, and the number of replicates are provided in Supplementary Table S1.
